# Control of the Nucleotide Cycle in Photoreceptor Cell Extracts by Retinal Degeneration Protein 3

**DOI:** 10.3389/fnmol.2018.00052

**Published:** 2018-02-21

**Authors:** Hanna Wimberg, Ulrike Janssen-Bienhold, Karl-Wilhelm Koch

**Affiliations:** ^1^Biochemistry, Department of Neuroscience, University of Oldenburg, Oldenburg, Germany; ^2^Department of Neuroscience, Visual Neuroscience, University of Oldenburg, Oldenburg, Germany

**Keywords:** RD3 protein, guanylate kinase, retinal dystrophy, cyclic nucleotide, phototransduction

## Abstract

Retinal degeneration protein 3 (RD3) is crucial for photoreceptor cell survival and linked to Leber Congenital Amaurosis type 12 (LCA12), a hereditary retinal disease in humans. RD3 inhibits photoreceptor guanylate cyclases GC-E and GC-F and is involved in transport of GCs from the inner to the outer segments. Otherwise, its role in photoreceptor physiology is poorly understood. Here, we describe a new function of RD3. Purified RD3 evoked an increase in guanylate kinase activity, an enzyme that is involved in the nucleotide cycle in photoreceptors. We demonstrate a direct interaction between guanylate kinase and RD3 using back-scattering interferometry and show by immunohistochemistry of mouse retina sections that RD3 and guanylate kinase co-localize in photoreceptor inner segments and to a lesser extent in the outer plexiform layer. Our findings point toward a more complex function of RD3 in photoreceptors. The RD3 – guanylate kinase interaction may also play a role in other cellular systems, while the GC – RD3 interaction is exclusive to photoreceptors.

## Introduction

Retinal dystrophies are heterogeneous disorders that typically result in the degeneration of photoreceptors leading to severe impairment or loss of vision. Different retinal disease forms include retinitis pigmentosa (RP), cone-rod or cone dystrophies (CORD/COD), Leber congenital amaurosis (LCA), color vision defects, night blindness and others (for a comprehensive review see Berger et al., 2010). Among these hereditary diseases, LCA is a particularly severe form of retinal degeneration causing blindness after birth or in the 1st year of life. At least 15 affected genes were identified in patients suffering from LCA and gene encoded proteins are involved in retinoid metabolism, retinal development and diverse cellular functions of photoreceptor cells. Examples of proteins associated with LCA are for instance RPE65 (retinoid isomerase), guanylate cyclase (abbreviated GC-E, RetGC1 or ROS-GC1) and RD3 (retinal degeneration 3 protein) (Perrault et al., 2000; den Hollander et al., 2008; Cideciyan, 2010; Preising et al., 2012). Mutations in *GUCY2D* (coding for photoreceptor specific guanylate cyclase type 1, GC-E) cause symptoms summarized under different disease types LCA1, CORD, and COD, which very often cause a drastic disturbance of the cGMP signaling system in rod and cone photoreceptor cells (Sharon et al., 2017). Excitation and adaptation of rod and cone cells is characterized by a fine-balanced homeostasis and interplay of 2 second messengers, cytoplasmic cGMP and Ca^2+^ (Koch and Dell’Orco, 2015). This cGMP signaling system is further impaired by mutations in *GUCA1A* coding for the Ca^2+^-sensor protein GCAP1, which is an essential regulator of GC-E activity (Palczewski et al., 1994; Frins et al., 1996; Olshevskaya et al., 2012).

Mutations in *RD3* of human patients correlate with the phenotypical characteristics of LCA12. Genetic screening approaches in worldwide collaborative efforts uncovered deletion and missense mutations in *rd3*, which were predicted to result in complete loss of function (Friedman et al., 2006; Perrault et al., 2013). Immunoprecipitation showed that RD3 binds to photoreceptor specific guanylate cyclases GC-E and GC-F and both proteins are not detectable in rods and cones of RD3 deficient mice (Azadi et al., 2010). It was further demonstrated that RD3 recruits GC-E to intracellular vesicles mediating GC-E trafficking from the endoplasmic reticulum to endosomal vesicles using COS-7 cells transfected with RD3 and GC-E (Azadi et al., 2010). This role of RD3 in correct GC-E trafficking and localization was recently confirmed by delivering *rd3* into a RD3 deficient mice strain by subretinal injection (Molday et al., 2013) and in a further study showing that several LCA1 causing mutations in GC-E disrupt the association with RD3 (Zulliger et al., 2015).

RD3 does not only bind to GC-E, but can act as an allosteric modulator inhibiting GC-E activity. It acts as an inhibitor of GC-E activity in two ways (Peshenko et al., 2011, 2016) by inhibiting the basal GC activity in a non-competitive manner (only decrease in *V*_max_) and decreasing the GCAP mediated activity in a competitive mode. Inhibition of GC-E is lost in a C-terminally truncated RD3 mutant, which resembles the mutated RD3 form that is associated with LCA12 (Peshenko et al., 2011). Transgenic mice lacking both photoreceptor GCs (GC-E and GC-F) undergo a degeneration of rods and cones (Baehr et al., 2007), comparable to RD3 deficient mice, pointing to analogous mechanisms of photoreceptor degeneration in LCA1 and LCA12 patients. The main conclusion from these studies is that correct trafficking and incorporation of GC-E in photoreceptor outer segments is essential for cell survival and that interaction of RD3 with GC-E is a crucial requirement for these processes. Furthermore, cones of GC-E knockout mice lack essential proteins of the phototransduction cascade like for example phosphodiesterase PDE6 and rhodopsin kinase GRK1 indicating a general disturbance of transport processes in cone cells (for a review see Karan et al., 2010).

Constitutive expression of RD3 was found in different mouse and human tissues including brain, kidney, liver, and spleen (Khan et al., 2015). The same authors further showed that RD3 loss in a mouse model correlates with an aggressive neuroblastoma cancer. These findings are, however, in disagreement with a previous study showing that inactivation of both RD3 alleles in LCA12 patients does not correlate with extraocular symptoms (Perrault et al., 2013). Recently, RD3 immunoreactivity was detected in normal human cell types, in particular in epithelial cells (Aravindan et al., 2017).

In the current project, we started to investigate the regulatory impact of RD3 on the cGMP signaling unit consisting of photoreceptor guanylate cyclases and GCAPs. During progression of the work, we detected that RD3 interacts at different steps within the nucleotide cycle in photoreceptor cells, which is important for maintaining a sufficiently high substrate level for the GCs. It has not only an inhibitory function toward GC-E activity, but a strong activating effect toward guanylate kinase (GUK), the enzyme that phosphorylates 5′-GMP to GDP. GUK is an essential, ubiquitous enzyme that catalyzes the first step for GTP recovery in the cell (Gaidarov et al., 1993). The subsequent phosphorylation steps are catalyzed by non-specific nucleoside diphosphate kinases. In this way these enzymes regulate the supply of nucleotides to different cellular processes like protein synthesis or vesicular trafficking (Gaidarov et al., 1993; Brady et al., 1996). Hall and Kühn (1986) purified GUK from bovine rod outer segments (ROS) and investigated GUK properties showing that it is present in small amounts (1 copy per 800 rhodopsin molecules), but is highly active. Our results show a new feature of GUK regulation by pointing to a new more complex role of RD3 in photoreceptor cell function.

## Materials and Methods

### Cloning of RD3 – His_6_ Construct

*Rd3* was cloned into a pETM-11 vector fused to a His_6_-Tag for expression in *Escherichia coli* (BL21+). The cDNA of human RD3 was amplified via polymerase chain reaction (PCR) using the following primer: 5′-GACTCCATGGGAATGTCTCTCATCTCATGGCT-3′ (Forward), 5′-TGACGGTACCTCAGTCGGCTTTGGGCGCC-3′ (Reverse). Vector and PCR product were cut by NcoI and KpnI. The Dephos & Ligations Kit (Merck, Darmstadt, Germany) was used for vector dephosphorylation and ligation. The vector including *rd3* was used for transformation in *E. coli* cells (XL1 blue, BL21+).

### Heterologous RD3 Expression and Purification via Ni-NTA

A 5 ml preculture of *E. coli* BL21+ cells - containing the pet-M11 RD3 construct - was grown overnight in LB-Medium at 37°C. The following day a 500 ml main culture was inoculated with the preculture until the OD_600_ reached 0.6. Expression was induced by adding 1 mM isopropyl-thiogalactoside (IPTG). The culture was incubated for 4 h at 37°C and 180 rpm. Cells were harvested at 5000 × g for 10 min at 25°C. Cell pellet was resuspended in 20 ml 50 mM Tris/HCl pH 8.0. Cell lysis was performed using 100 μg/ml lysozyme and 5 U/ml DNase while incubating 30 min at 30°C in a water bath. After adding 1 mM DTT and 0.1 mM phenylmethyl-sulfonylfluoride (PMSF) cells were centrifuged 1.5 h at 50000 × *g* and 4°C. Pellet containing the main portion of RD3 was homogenized in buffer 1 [20 mM phosphate buffer pH 7.4, 8 M urea, 10 mM imidazole, 500 mM NaCl, 5 mM β-mercaptoethanol (β-ME), 1 mM PMSF] and incubated overnight at 4°C. Suspension was centrifuged 60 min at 50000 × *g* and 4°C. The supernatant was applied to a Ni-NTA column (column volume CV: 20 ml) equilibrated with buffer 1. After sample application the column was washed with 3 CV buffer 1. Refolding of the protein was performed using a gradient for stepwise exchange to buffer 2 (20 mM phosphate buffer pH 7.4, 10 mM imidazole, 500 mM NaCl, 5 mM β-ME, 1 mM PMSF, 1 mM histidine, 10% glycerol) in 5 CV (flow: 1 ml/min). The protein was eluted in 2 CV of buffer 3 (20 mM phosphate buffer pH 7.4, 500 mM Imidazole, 20 mM histidine, 500 mM NaCl, 5 mM β-ME, 1 mM PMSF). Fractions (1 ml) were collected and analyzed via standard sodium dodecyl sulfate polyacrylamide gel electrophoresis (SDS-PAGE).

### GC Activity Assay

Biological function of purified RD3 was tested in a GC assay by monitoring its inhibitory impact on photoreceptor GC activity. Stable transfected HEK-293T cells were used to express recombinant human GC-E and GC-F and prepared as described earlier (Zägel et al., 2013). Alternatively, bovine ROS were prepared from fresh bovine eye balls obtained from a local slaughterhouse according to an established standard procedure (Koch et al., 1994). Soluble extracts were obtained from ROS as described below (PDE-activity assay).

Human and bovine GCAP1 and GCAP2 were expressed and purified in *E. coli* as described before (Hwang et al., 2003; Helten and Koch, 2007; Sulmann et al., 2017). The expression of GC-E and GC-F in HEK-293T cells was confirmed via western blot (Helten et al., 2007). For the guanylate cyclase activity assay 10 μl of GC-E or GC-F containing HEK-293T cell membranes were incubated with 5 μM GCAP, 2 mM K_2_H_2_EGTA and varying RD3 concentrations (final volume: 30 μl) for 5 min at room temperature. 20 μl GC buffer (75 mM Mops/KOH pH 7.2, 150 mM KCL, 10 mM NaCl, 2.5 mM DTT, 8.75 mM MgCl_2_ 2.5 mM GTP, 0.75 mM ATP, 0.4 mM Zaprinast) were added and the reaction was started for 5 min at 30°C. Reaction was stopped by adding 50 μl 100 mM EDTA. Levels of produced cGMP were determined by reversed phase HPLC (Koch and Helten, 2008).

### PDE6 – Activity Assay

All steps were done on ice and under dim red light conditions. 1 ml ROS (3 mg/ml) was centrifuged for 10 min at 125000 × *g* and 4°C. The pellet was resuspended in 750 μl buffer 4 [20 mM BTP (pH 6.9), 120 mM KCl, 0.2 mM MgCl_2_, 5 mM DTT]. Pellet was homogenized and 5 mM MgCl_2_ were added. The suspension was centrifuged for 30 min at 80000 × *g* and 4°C. Pellet was resuspended in buffer 5 [5 mM Tris/HCl (pH 6.9), 5 mM DTT]. The rhodopsin concentration was set to 1 mg/ml. The sample was exposed to light for 10 min and again centrifuged for 30 min at 80000 × *g* and 4°C. The supernatant was used for the PDE activity measurement. 10 μl PDE enriched solution were mixed with 15 μl reaction buffer [10 mM Hepes (pH 7.4), 100 mM NaCl, 2 mM DTT, 2 mM MgCl_2_], 2.5 μl 10 mM cGMP, and the volume was adjusted to 50 μl. 1 μM RD3, RD3 elution buffer or water were added. For complete PDE activation 10 μl trypsin (1.5 mg/ml) were added. Samples were incubated for 10 min at 25°C and reaction was stopped by adding 50 μl 100 mM EDTA and incubation for 5 min at 95°C. The samples were analyzed by HPLC and 5′-GMP levels were determined.

### Spectrophotometric Assay

Guanylate kinase activity upon increasing RD3 concentrations was measured via a spectrophotometric assay (Hall and Kühn, 1986). The phosphorylation of 5′-GMP to GDP was monitored using a coupled enzyme assay. The decrease in absorbance at 340 nm coupled to NADH oxidation to NAD+ was recorded for 10 min. The protocol and buffers were used as described by Hall and Kühn (1986). An amount of 0.02 U of the guanylate kinase (GUK from porcine brain, Merck, Darmstadt, Germany) per measurement was used. One unit will convert 1.0 μmole each of GMP and ATP to GDP and ADP per min at pH 7.5 at 30°C. RD3 concentrations varied from 0 nM to 3.5 μM. The measurement for each concentration was done in triplicates and was repeated independently three times with similar results. For determination of enzyme kinetics (*V*_max_ and *K*_M_) we varied the concentration of the substrate GMP at a constant ATP (2 mM) and enzyme concentration (0.77 μg GUK per 1 mL of sample volume).

### RD3 Crosslinking

The crosslinker BS^3^ (Thermo Fisher Scientific, Waltham, MA, United States) was used to determine the RD3 status in solution. BS^3^ is able to crosslink proteins that are in close proximity of 11.4 Å. RD3 (0.1 μg) and BS^3^ (0.5 mM) were incubated for 2 min at room temperature. The reaction was stopped by adding 4 μl of 1 M Tris-HCl (pH 7.5). To detect a possible binding to GUK, lysates from normal HEK cells and a stable GUK expressing HEK cell line were incubated with RD3 and BS^3^. Samples were analyzed via SDS-PAGE and immunoblotting.

### Retina Preparation and Subcellular Fractionation

Experiments were conducted with C57 BL/6I J mice (age 3–6 months). All experiments were approved by the local animal welfare committee (LAVES, *Niedersächsisches Landesamt für Verbraucherschutz und Lebensmittelsicherheit*) and followed the guidelines of the German Animal Welfare Act (*Tierschutzgesetz; BGBl. I S. 1206, 1313 and BGBl. I S. 1934*). Mice were killed by cervical dislocation after being deeply anesthetized with CO_2_. Eyes were enucleated and either eyecups or isolated retinas were dissected in physiological PBS, pH 7.4. Eyecups were fixed in 4% PFA, 3% sucrose in 0.1 M PB, pH 7.4 for 20 min, washed in 0.1 M phosphate buffer (PB), pH 7.4 and subjected to cryoprotection (30% sucrose in 0.1 M PB, pH 7.4) overnight at 4°C. Eyecups were embedded in Tissue-Tek and 20 μM thick sections were cut.

For subcellular fractionation, isolated retinas (*n* = 8) were pooled and homogenized in homogenization buffer (HP, 70 μl per retina) containing 50 mM Tris/HCl, pH 7.4, 2 mM ethylene glycol-bis(2-aminothylether)-N,N,N′,N′-tetraacetic acid (EGTA), 2 mM EDTA, 0.1 mM sodium orthovanadate, 1 mM 1,4-dithioerythritol, 1 mM PMSF, 2 μg/ml leupeptin, 5 μg/ml aprotinin, protease (complete mini, EDTA-free, Roche, Basel, Switzerland) and phosphatase inhibitor cocktail (PhosphoSTOP, Roche, Basel, Switzerland). This sample was named as total homogenate (Th). The Th fraction was further processed and centrifuged for 10 min at 1300 rpm and 4°C. The supernatant was transferred into a new tube and the pellet again dissolved in HP-buffer and centrifuged for 10 min at 1300 rpm and 4°C. The supernatants were pooled and further centrifuged for 60 min at 14000 rpm and 4°C. The resulting pellet was termed as P fraction representing the crude retinal membrane fraction, whereas the supernatant was termed S fraction standing for crude retinal cytosolic fraction.

Via immunoblotting these retina fractions (Th, P, and S) were probed with primary antibodies directed against GUK [GUK1 (H-77) rabbit polyclonal, (Santa Cruz Biotechnology, Dallas, TX, United States; dilution 1:1000] and RD3 [RD3 (B-8) mouse monoclonal, Santa Cruz Biotechnology, Dallas, TX, United States; dilution 1:500] according to established laboratory protocols (Helten et al., 2007). Of each retina fraction (Th, P, and S) 30 μg total protein were separated on 7.5% SDS-gels and proteins were transferred by semi-dry blotting onto PVDF membranes. For immunodetection of GUK on PVDF membranes the transferred proteins had to be fixed with 0.5% glutaraldehyde in PBS for 20 min.

### RNA Isolation, cDNA Synthesis

Retinae from mice of the C57 Bl6 J strain (adult, 2–3 months) were dissected in sterile PBS. The “Total RNA Isolation Kit Nucleo Spin RNAXS” (Macherey-Nagel, Düren, Germany) was used to isolate RNA. RNA concentration was measured via Nanodrop and 1 μg of RNA was DNAse treated. Samples for cDNA synthesis were prepared: 1 μl Oligo(dt)_15_ Primer (500 μg/ml), 1 μl Random Primer (500 μg/ml), 1 μl Oligonucleotide dNTP Mix (10 mM per nucleotide), 5 min 65°C, 1 min on ice. In a second step 1 μl RNase-inhibitor (40 U/μl), 4 μl 5x First Strand Buffer, 1 μl 0.1 M DTT and 1 μl SuperScript^TM^ III Reverse Transcriptase (200 U/μl) were added, followed by incubation at 25°C (5 min), 50°C (50 min), and 7°C (15 min).

The primer used for cDNA amplification of GUK and RD3 sequences were the following:

GUK1: 5′-GTCGGGTCCCCGCGGACGG-3′ and 5′-CAGGCGTGGCCAGTTCCCTGT-3′
GUK2: 5′-GGAGGCTGCAACACATCAAGTAG-3′ and 5′-CAGGCGTGGCCAGTTCCCTGT-3′
RD3: 5′-CCTCATCCCGTGGCTCCGGTG-3′ and 5′-CGCCCTGAACTCCGGCATGC-3′

Polymerase chain reaction to verify the presence of mouse GUK1, GUK2, and RD3 was done in parallel with an actin control. The PCR products were purified from the agarose gel and sequenced (GATC Biotech, Konstanz, Germany).

### Immunohistochemistry and Image Aquisition

Mouse retinal sections were washed with TBS buffer, (50 mM Tris/HCl, 100 mM NaCl), pH 7.4. Blocking was performed using 10% donkey serum in TBS containing 0.2% Triton X-100. All antibodies were diluted in TBS containing 2.5% donkey serum and 0.1% Triton X-100. To get optimized immunoreactivity patterns in double-labeling experiments, serial incubations were performed. Therefore, incubation was first carried out with the primary antibody directed against RD3 (mouse monoclonal B-8, Santa Cruz Biotechnology, Dallas, TX, United States, diluted 1:50, overnight at 4°C), followed by the first set of secondary antibodies (donkey anti mouse IgG conjugated to Alexa-488, Invitrogen, diluted 1:1000), which were applied for 2 h at room temperature. Following several washing steps with TBS, pH 7.4, sections were subsequently incubated with the anti-GUK antibodies (rabbit polyclonal H-77, Santa Cruz Biotechnology, Dallas, TX, United States, diluted 1:100, overnight at 4°C), washed again and finally incubated with the second set of secondary antibodies (donkey anti-rabbit IgG conjugated to Alexa-568, Invitrogen, diluted 1:1000) for 2 h at room temperature.

Images were acquired with a confocal laser scanning microscope (Leica TCS SP8). Stacks (12 slices, z-distance 300 nm) of retinal sections were scanned with an HC PL APO CS2 63x/1.4 oil objective at a resolution of 1024 pixels × 1024 pixels. To rule out crosstalk between channels scans of different wavelength were performed sequentially. Images are either presented as projections of 12 × 0.3 μm scans or single scans (0.3 μm). To assess co-localization single scans were superimposed and image processing (adjustment of brightness and contrast) was performed using Adobe Photoshop CS3 (Adobe Systems, San Jose, CA, United States).

### Back Scattering Interferometry (BSI)

Back scattering interferometry is a powerful technique to investigate protein – protein interaction. Label-free samples can be measured in solution making use of a changing interference pattern expressed in relative phase change. Details of instrumentation set up and performance were described recently (Markov et al., 2004; Bornhop et al., 2007; Kussrow et al., 2009; Sulmann et al., 2017).

Sample preparation – HEK-293T cells containing GUK were harvested, resuspended (20 mM phosphate buffer pH 7.0, 150 mM NaCl and 0.005% Tween 20) and lysed by using a syringe with a 0.7 mm tube. Cell suspension was centrifuged for 10 min at 13000 rpm. The final concentration of HEK/GUK cell suspension in the assay was 0.1 μg/μl and was obtained from cell lysates of stable GUK transfected HEK293 cells. RD3 was supplied in the same cell suspension buffer and its concentration varied from 0 to 300 nM. HEK-293T cell lysate not expressing GUK was used as a control and also titrated with RD3. Titration series were repeated with TEV protease that served as a His_6_ tagged control protein to exclude possible non-specific interactions arising from stretches of His residues. Samples were incubated overnight at 4°C to reach equilibrium. Prior to use each sample was briefly sonicated for 5 min (sonication bath, 80W).

## Results

### Functional RD3 Controls the Nucleotide Cycle in Photoreceptor Cells

This study started by testing the role of RD3 interacting with GC-E (Azadi et al., 2010; Peshenko et al., 2011). For this purpose we needed to express and purify recombinant human RD3, which was described to be quite challenging, because the protein tends to easily precipitate (Peshenko et al., 2011). Thus, we decided on cloning a His_6_-tagged variant and tested its functionality via the effect on human GC-E.

**FIGURE 1:**
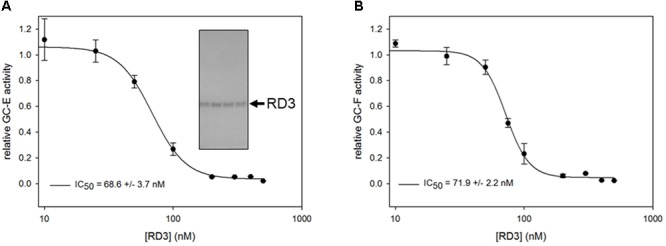
Inhibition of GC-E **(A)** and GC-F **(B)** wild-type expressed in HEK-293T cells by purified RD3. GC-E (left panel) and GC-F (right panel) were incubated with GTP containing buffer at 20 nM Ca^2+^, 5 μM GCAP1 and increasing concentrations of RD3 (0–500 nM). Each incubation with RD3 was measured in triplicates. Data are shown as mean ± SD, curve fitting was performed with SigmaPlot 13 using a normal Hill equation. GC activities were determined by the level of produced cGMP analyzed by HPLC. Inset: Coomassie stained SDS-gel with fractions containing purified RD3.

Within 4 h after the induction of protein expression in *E. coli*, a high amount of RD3 is produced becoming visible as an intense band around 26 kDa in a polyacrylamide gel (data not shown). Since the main fraction of the protein is found in inclusion bodies, RD3 was extracted from the *E. coli* pellet by an 8 M urea containing buffer. Extracted protein bound to a Ni-NTA column via its His_6_-tag and was refolded directly on the column with a buffer exchange step. It eluted from the column in a buffer containing competitive amounts of histidine and 10% glycerol for protein storage at -80°C. Fractions containing RD3, but with impurities were discarded, fractions showing a high grade of purity in a Coomassie blue stained SDS-gel (inset in **Figure 1**) were used for subsequent protein assays. The protein stored in 10% glycerol proved to be stable and even showed sufficient activity in a GC activity-assay after a couple of months.

A critical test for the functional state of RD3 is evaluating its role as an effective inhibitor of mammalian GC-E (Peshenko et al., 2011), which we tested via a standardized GC-E – assay under activating conditions (adding 5 μM recombinant human GCAP1 at low Ca^2+^ concentration). RD3 inhibited GC-E activity completely above 150 nM (**Figure 1A**). Inhibition was half maximal at about 70 nM in agreement with previous observations (Peshenko et al., 2011). Further, we tested the inhibition of GC-F activity, the second photoreceptor specific GC form, yielding nearly identical results (**Figure 1B**). Results were reproducible in independent repetitions of the experiment. Using bovine GCAP1 instead of the human form did not change the results. Thus, the His-tagged RD3 is functional and suitable for further investigations.

We used two different sources for testing inhibition by RD3. One was recombinant GC-E and GC-F present in membranes from HEK-293T cells as in **Figure 1**, and a second approach was employing native GC-E and GC-F present in bovine ROS membranes (Helten et al., 2007). By analyzing nucleotide profiles of incubations with native GCs in washed ROS membranes by an HPLC assay, we observed substantial changes in GDP and 5′-GMP levels, when RD3 was present in the incubations mixture (Supplementary Figure S1). Based on previous observations made in our laboratory, washed ROS membranes used for GC assays lack most of cytosolic, i.e., soluble proteins, but are not completely free of them. Therefore, these changes could in principle originate from effects of RD3 on phosphodiesterase 6 (PDE6) or guanylate kinase (GUK). Subsequent tests showed no effect on PDE6 (Supplementary Figure S2A), but on GUK. We also tested, whether RD3 might have relieved the inhibition of PDE by zaprinast that is routinely present in the GC assay buffer, but we did not find any effect (Supplementary Figure S2B).

### A Spectrophotometric Analysis of GUK Activity Showed an Increase in GDP Production by RD3 Interaction

**FIGURE 2:**
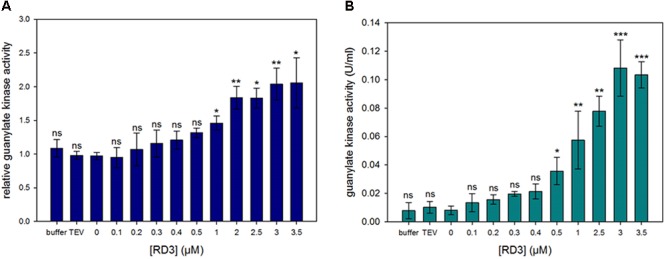
Guanylate kinase activity assay. Activity determination of GUK in a spectrophotometric assay. The 5′-GMP to GDP conversion is coupled to NADH oxidation and the decrease in absorption is measured at 340 nm. Effect of varying RD3 concentrations on enzyme activity was tested. GUK in a soluble extract from bovine ROS **(A)** and GUK from porcine brain **(B)** were used. Normalization value 1.0 in **(A)** corresponds to GUK activities in the absence of RD3, which were between 0.025 and 0.042 U/mg rhodopsin. The experiment was repeated independently three times with two to three replicates for each sample yielding similar results. Statistical analysis was performed using GraphPad Prism V5.01 (GraphPad Prism Inc.). The data failed the Kolmogorov–Smirnov normality test. Therefore, the Kruskal-Wallis one-way ANOVA with *post hoc* tests (Dunn’s Multiple Comparison) were applied to determine if groups were significantly different from each other (significance levels are ns = *p* > 0.05, ^∗^*p* ≤ 0.05, ^∗∗^*p* ≤ 0.01, ^∗∗∗^*p* ≤ 0.001).

Hall and Kühn (1986) used a photometer based coupled enzyme assay to measure the activity of guanylate kinase from ROS preparations, which we employed in the following to measure GUK activity in our ROS preparations. Further, we used a commercially available sample of GUK from porcine brain in the assay to compare it with the activity of endogenous GUK in our ROS preparations. Incubation of GUK with its substrate 5′-GMP was done in the presence and absence of RD3 (concentrations of RD3 ranging from 0 to 3.5 μM). The kinase activity increased with increasing concentrations of RD3 (**Figure 2**). This effect was visible with samples from ROS preparations (A) and even stronger with commercial GUK (B). Controls containing only the RD3 elution buffer or another protein of similar size, a His_6_-tagged TEV (Tobacco Etch Virus) protease showed no increase in GUK activity.

A nearly tenfold increase in GDP production was seen with saturating concentrations of RD3 near 3 μM. GUK from porcine brain evoked an increase in kinase activity of about 10 times (B). For the GUK in ROS preparations a doubling of the activity could be measured (A). A clear effect on the stimulation of GUK activity was already evident at 300 nM, but we observed no saturation up to 3 μM RD3. Kinetic analysis revealed that the *V*_max_ increased from 0.014 to 0.045 U/mL when 500 nM RD3 was added, but the *K*_M_ slightly increased as well from 2.01 to 2.74 μM (for substrate GMP). Calculating *k*_cat_/*K*_M_ showed an increase from 3.3 × 10^6^ to 7.8 × 10^6^ M^-1^s^-1^, which is in good agreement with the value of 10^7^ M^-1^s^-1^ reported by Hall and Kühn (1986) for GUK in ROS. Attempts to increase the RD3 concentration in the assay medium failed due to the propensity of RD3 forming aggregates in concentration devices (e.g., centrifugal filter units). The lower degree of GUK activation in ROS extracts is probably due to differences in ROS preparations with varying amounts of GUK present.

### GUK and RD3 Are Expressed in the Mouse Retina and Can Both Be Localized in the Inner Segments of Photoreceptor Cells

It is unknown, whether RD3 and GUK co-express and co-localize in the mammalian retina. Therefore, we tested mouse retina tissue for expression of both proteins on different levels, namely identification of transcripts by reverse transcriptase PCR, subcellular fractionation and immunoblotting and immunohistochemistry.

**FIGURE 3:**
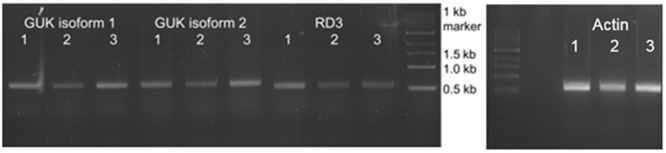
Analysis of GUK and RD3 expression in mouse retina tissue. Reverse transcription of RD3 and GUK analyzed by agarose gel electrophoresis. RNA was extracted from three mouse retina samples (1–3) followed by reverse transcription into cDNA. PCR products obtained with GUK and RD3 specific primers are shown. Exact lengths of transcripts are: GUK isoform 1, 600 bp; GUK isoform 2, 621 bp; RD3, 585 bp. RNA quality check was done with intron-spanning actin primers, indicating no contamination of the samples with genomic DNA, and PCR products were sequenced for correctness.

RNA was extracted from three mouse retina preparations. Database research (NCBI) showed the existence of two GUK isoforms in mouse. Isoform 1 (NP_032219.2), transcript variant 1 (NM_008193.3) represents the longer variant of 1098 base pairs (219aa). Isoform 2 (NP_001152882.1), transcript variant 2 (NM_001159410.1) has a shortened *N*-terminus resulting in 935 bp (207aa). Specific primers to recognize both isoforms were designed. After PCR with GUK (isoforms 1 and 2) and RD3 specific primers a transcript of ∼600 bp was detected (**Figure 3**) for each molecule. RNA quality check was done with an actin control and intron spanning primers, not showing a contamination with genomic DNA. To verify the result, PCR products were sent for sequencing (GATC Biotech, Konstanz, Germany). Sequence analysis confirmed the PCR products as transcripts of GUK isoforms 1 and 2 and RD3, respectively.

**FIGURE 4:**
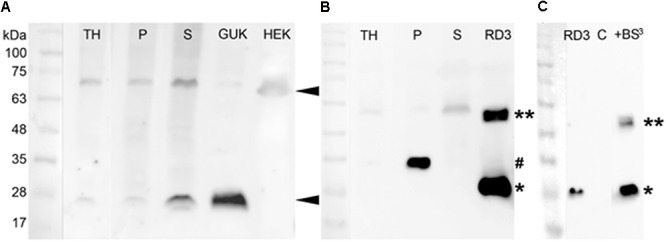
Detection of GUK and RD3 in mouse retina tissue by specific antibodies and RD3 crosslinking. **(A)** Primary anti-GUK antibody (diluted 1:1000) was tested on mouse retina fractions (30 μg total protein amount) and stable GUK expressing HEK-293T cells (15 μg total protein amount). GUK-immunoreactive proteins are marked ◄. **(B)** Anti-RD3 antibody (diluted 1:500) was tested on mouse retina fractions (30 μg) and RD3 expressed in *Escherichia coli* (1 μg). RD3-positive proteins in retinal fractions were marked (^#^), as well as the monomeric (^∗^) and dimeric (^∗∗^) form of purified RD3. TH, total retina homogenate; P, crude retinal membrane faction; S, crude retinal cytosolic fraction (see section “Materials and Methods” for details). **(C)** For the crosslinking experiment, the same anti-RD3 mouse monoclonal antibody was used (diluted 1:1000). An amount of 0.1 μg RD3 and 7.5 μg HEK cell lysate (C = negative control) were used. RD3 immunoreactive bands were detected at 26 and 55 kDa. The second band (marked with ^∗∗^) correlates with a possible crosslinked product.

Although this result confirmed the presence of GUK and RD3 in mouse retina preparations on the transcript level, we needed to detect the expressed proteins via western blotting in retinal tissue. Further, we needed to verify the specificity of GUK and RD3 antibodies for immunostaining. Therefore, a stable HEK-293T cell line expressing mouse GUK (isoform 1) was created for the use as positive control. As a negative control for testing non-specific crossreactivities HEK-293T cell lysate was used. The supernatant of lysed GUK expressing HEK-293T cells showed a distinct band at about 25 kDa, which is consistent with the expected theoretical molecular mass of 22 kDa (**Figure 4A**, marked with an arrow head). No signal at this electrophoretic mobility was detected in the negative control. Mouse retinae were separated into membrane and cytosolic fractions and probed by western blotting for the presence of GUK and RD3 (**Figure 4**). GUK was detected at the same molecular mass of ∼25 kDa like in the positive control. The signal intensity of GUK, however, was stronger in the cytosolic fractions (S) than in the membrane fraction (P). In addition to the band at 25 kDa, a second band around 70 kDa crossreacted with the anti-GUK antibody, which was stronger in the retina fractions than in the positive GUK control, but was also present in the negative control (HEK, marked with an arrow head).

The band at 70 kDa may represent a multimeric GUK variant because the signal intensity at 25 kDa in the P and S samples correlated with those of the GUK signal in these fractions. However, because a diffuse signal at around 70 kDa was also detected in the HEK cell negative control, we cannot fully exclude any unspecific binding.

For the detection of RD3 in mouse retina fractions, human RD3 purified from *E. coli* was used as a positive control and showed a clear signal at around 28 kDa (**Figure 4B**, marked with an asterisk). The antibody detected a signal of slightly higher molecular mass at 33 kDa in the membrane (marked with #), but not in the cytosolic fraction of mouse retinae. This may be due to modifications of RD3 in native tissue. Probing recombinant RD3 with the anti-RD3 antibody, however, yielded a second band at about 55 kDa (marked with ^∗∗^ in **Figure 4B**). We reasoned this to be a dimeric form of RD3 and tested this assumption by a crosslinking experiment (**Figure 4C**). We crosslinked purified RD3 using the crosslinker BS^3^, which can crosslink proteins in close proximity at a distance of 11.4 Å. Crosslinked products were analyzed by SDS-PAGE and subsequent western blotting. While monomeric RD3 was detected in the presence and absence of crosslinker BS^3^ (^∗^), a signal at 55 kDa appeared after crosslinking (^∗∗^) indicating that RD3 can be present as a dimer in solution. As negative control (C) we used 7.5 μg of HEK-293T cell lysate.

A second band of slightly higher molecular mass at 58 kDa was also visible in the retina fractions, especially in the cytosolic fraction (S) pointing toward an enriched concentration of dimeric RD3 in the cytosol. A third quite weak signal of ∼90 kDa was detected in all retina fractions and in the positive control. We interpreted this to be an oligomeric or aggregated RD3 variant.

The results indicate that RD3 and GUK can both be detected in retinal tissue on the transcript and protein level. It seems that GUK is mainly present in the cytosol of the retina, whereas RD3 is mainly present in the membrane (P) fraction. This observation is consistent with reports showing that RD3 is connected to vesicular trafficking in photoreceptors (Azadi et al., 2010; Zulliger et al., 2015).

**FIGURE 5:**
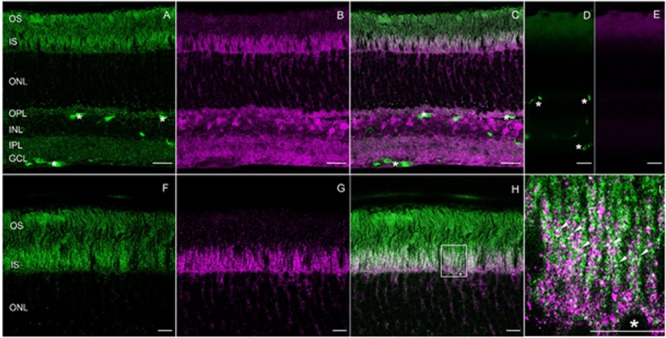
Expression and co-localization of RD3 and GUK in wild-type mouse retina. Cryosections of mice retinae were stained for RD3 (green, **A,C,F,H,I**) and GUK (magenta, **B,C,G–I**). The overlays **(C,H,I)** reveal partial co-localization of the two proteins in the inner segments of photoreceptors, particularly visible at higher magnification (**I**, arrow heads). For orientation a photoreceptor soma is labeled by an asterisk (^∗^, **H,I**). Control stainings with secondary antibodies are shown in **(D,E)**. Unspecific staining of blood vessels is indicated (^∗^ in **A,C,D**). **(A–E)** Maximum projections of confocal scans (12 optical sections, 0.3 μm thick). **(F–H)** Single scans, 0.3 nm thick. Scales: 20 μm **(A–E)**, 10 μm **(F–I)**.

To localize RD3 and GUK in the retina and to determine a possible co-localization, mouse retina cryosections were stained with the tested antibodies. The results showed a localization of RD3 (green) in the photoreceptor inner (IS) and outer segments (OS) and to a lesser extent in the outer plexiform layer (OPL) (**Figure 5A**). GUK-immunoreactivity (magenta) revealed a more complex distribution pattern and was localized in the IS, outer nuclear layer (ONL), OPL, inner plexiform layer (IPL), and ganglion cell layer (GCL) (**Figure 5B**). Thus, except for the OS of the photoreceptors, GUK was detected in most layers of the retina a finding that is consistent with the general housekeeping role and ubiquitous mRNA expression of GUK (Brady et al., 1996). The overlay of both immunoreactivity patterns (**Figures 5C,H,I**) revealed a partial co-localization of RD3 and GUK as indicated by the white signals, of which the vast majority was seen in photoreceptor inner segments (**Figure 5I**, arrow heads). The control staining of the secondary antibodies (**Figures 5D,E**) showed a weak back ground staining in the OS layer and a non-specific labeling of blood vessels for the donkey-anti-mouse antibody (**Figures 5A,D**, asterisk).

To our best knowledge, localization of GUK in the retina via immunohistochemistry has not yet been documented in the literature. But, a study on enzymes activity involved in phototransduction in different retinal layers was conducted, which also investigated the activity of GUK (Berger et al., 1980). The authors of this study proposed an enriched activity and presence of GUK in photoreceptor inner segments, a finding that is in accordance with the data presented here. RD3 localization in photoreceptor IS and OS was already stated by different studies (Azadi et al., 2010; Zulliger et al., 2015).

### RD3 and GUK Interact Directly as Monitored by Back Scattering Interferometry (BSI)

Control of GUK activity by RD3 and their co-localization in retinal layers indicated that both proteins might form a direct functional protein complex. Initial attempts to use surface plasmon resonance for studying the interaction of GUK and RD3 failed, because the immobilization of RD3 on the sensor chip surface probably led to aggregation. Therefore, we employed Backscattering interferometry (BSI) a label free binding assay taking place in free solution without need to immobilize one binding partner. It represents a powerful technique to study protein–protein interaction with a very high sensitivity. An induced conformation and hydration change can be detected in the alteration of the refractive index of the solution. Even the binding affinities of membrane associated proteins can be measured (Bornhop et al., 2007, 2016; Baksh et al., 2011).

**FIGURE 6:**
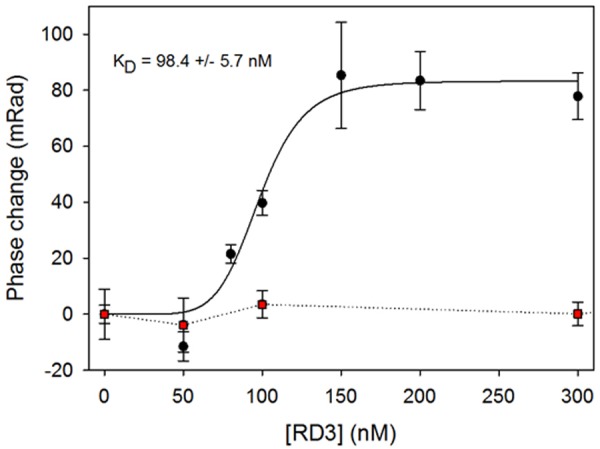
RD3 and GUK interaction detected by Back Scattering Interferometry (BSI). Various RD3 concentrations ranging from 0 to 300 nM were tested for interaction with GUK (black dots) by BSI. Titration of a control protein (TEV) to GUK showed no binding (red squares). Titration series is an example of three independent experiments. Each symbol is the mean ± SD of five data points. Curve fitting was performed with SigmaPlot 13 using a normal Hill equation.

We used purified RD3 and the supernatant of lysed HEK cells containing recombinant GUK. Measurements with the reference sample containing the same amount of HEK cell lysate, but not expressing GUK, were used in control titrations with the same amounts of RD3. Binding events by BSI are recorded as a phase change (change in an interference pattern), which we monitored as a function of RD3 concentration (**Figure 6**). Control recordings were subtracted and the resulting phase change pattern yielded an apparent *K*_D_ value of ∼100 nM. The control protein (TEV protease) showed no binding with GUK containing HEK-293T cell lysates (red squares in **Figure 6**). These results pointed toward a direct interaction of RD3 and GUK. The apparent affinity of RD3-GUK interaction appears higher than the efficacy with which RD3 controlled GUK activity (**Figure 2**). This discrepancy could arise from differences in the detection mode, a direct binding assay (BSI) versus a coupled enzyme assay utilizing several reactions and equilibria.

## Discussion

Loss of RD3 function is connected to retinal degeneration in humans and leads to Leber congenital amaurosis type 12 (LCA12) indicating a crucial role of RD3 in photoreceptor physiology. A nonsense mutation in *rd3* leads to a non-functional truncated protein variant causing the disease phenotype (Friedman et al., 2006). So far, two functions for RD3 are known. RD3 can inhibit the GC-E activity (Peshenko et al., 2011) and is involved in transporting processes from the photoreceptor inner segments to the outer segments (Azadi et al., 2010; Zulliger et al., 2015). To analyze further the characteristics of RD3, we were able to express and purify functional RD3 from *E. coli*. The protein inhibited the GC-E and GC-F activity in a nanomolar range with an IC_50_ value of about 70 nM. The results are in accordance with the report by Peshenko et al. (2011) showing that RD3 inhibits GC-E at nanomolar concentrations and making us confident to use purified RD3 for subsequent studies.

**FIGURE 7:**
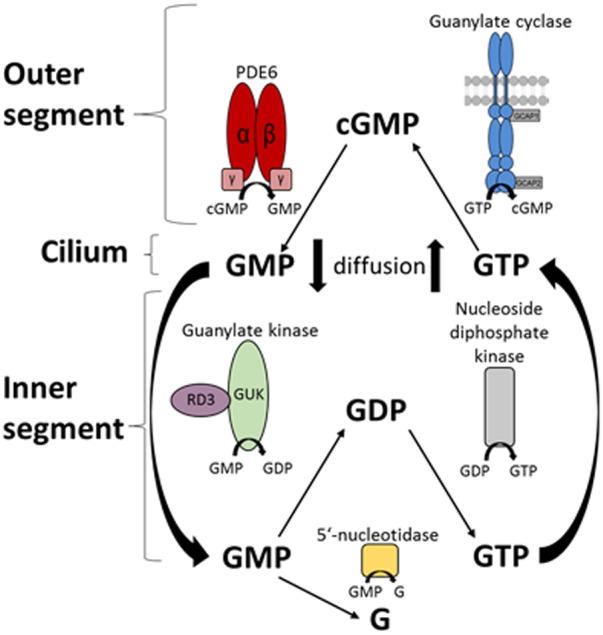
Schematic overview of the nucleotide cycle in photoreceptor cells. Cyclic GMP is the second messenger of photoreceptor excitation. After light-triggered degradation to 5′-GMP by the PDE6, 5′-GMP diffuses to the inner segment of the photoreceptor. Here it can be further degraded to guanosine or is recycled by the guanylate kinase (GMP → GDP) and the nucleoside diphosphate kinase (GDP → GTP). RD3 interacts with GUK and upregulates its activity. GTP then diffuses back to the outer segments, where the enzymes of the phototransduction cascade use it, specifically the GCs synthesizing cGMP from GTP.

The central finding of this study is that RD3 controls enzymes of the nucleotide cycle in photoreceptors, not only the final step of second messenger (cGMP) synthesis by GC-E, but also the important pre-step that is catalyzed by GUK, which provides a constant supply of the GC substrate GTP. By catalyzing the 5′-GMP to GDP conversion (**Figure 7**), GUK recycles the downstream product of the light-triggered phototransduction cascade 5′-GMP. Restoration of GTP levels is therefore critical for the dark state recovery in photoreceptors. Our model (**Figure 7**) implicates that 5′-GMP diffuses to the inner segments for the recycling of GTP, where GUK produces GDP under control by RD3. Nucleoside diphosphate kinase then forms GTP from GDP. Constant diffusion of nucleotides between photoreceptor compartments would ensure sufficient supply of enzyme substrates.

Previous reports and our data are in accordance with this model. For example, two earlier studies revealed the presence of RD3 in the OS, IS, OPL, and axon terminals of photoreceptor cells by immunohistological staining (Azadi et al., 2010; Zulliger et al., 2015). Similarly, we localized RD3 in the IS, OS, and OPL (**Figure 5**). Furthermore, we here show for the first time GUK immunohistochemical staining in a mammalian retina. GUK showed a widespread expression in different retinal layers except in OS, which is consistent with activity profiles of GUK that were measured in different retina layers by Berger et al. (1980). The double staining of GUK and RD3 further provides strong evidence that both proteins are present in rod photoreceptor IS, where they might form a functional complex. Our BSI recordings demonstrate a high affinity interaction of RD3 and GUK supporting our conclusion.

The acceleration of GTP recycling by RD3 may be a physiologically important step after strong illumination, which requires replenishment of an exhausted GTP pool. A fivefold increase of GUK activity was observed after illumination (Gaidarov et al., 1993), but the controlling factor has not yet been identified. Based on our results, RD3 seems to be the factor linking illumination and increase in GTP supply. However, other metabolic pathways could participate in replenishing or maintaining GTP levels. The citric acid cycle directly produces GTP. Furthermore, ATP is a GUK substrate and cellular ATP levels could have controlling effects. For example, ATP consumption is dependent on illumination and changes from dark to light by more than fourfold (Okawa et al., 2008). Photoreceptors use ATP differently in darkness and in light pointing to mutual dependencies of metabolic fluxes (Du et al., 2016). It will be interesting to learn in future studies how these regulatory steps are integrated on the cellular level.

So far, GUK was not identified as a candidate gene for retinal diseases. This may reflect the absence of a retina specific form of GUK. However, the GUK gene is localized on chromosome 1 and its gene locus overlaps with that of USH2A, which is linked to an autosomal form of Usher disease (Fitzgibbon et al., 1996). Future studies are needed to evaluate, whether GUK is involved in ciliary diseases like Usher. Interestingly, patients suffering from LCA1 (due to mutations in the gene for GC-E) show a less severe phenotype than LCA12 patients. Loss of RD3 function in LCA12 leading to dysregulation of GC-E and GUK activity may also explain why LCA1 and LCA12 phenotypes differ and why the latter is even more severe (Preising et al., 2012; Molday et al., 2014).

The guanylate kinase (GUK) is an essential ubiquitous enzyme involved in the nucleotide metabolism of cells. Therefore, it is involved in diverse cellular mechanisms and is used as a target for distinct therapeutic approaches. Next to its role as a housekeeping enzyme of the cell, GUK may also function as a tumor suppressor via its control of 5′-GMP and GTP levels (Gaidarov et al., 1993). Additionally, GUK is manifold used as a regulator in viral and cancer therapies. For example, 5′-GMP analogs serve as potent GUK inhibitors or are widely used as antiviral and anticancer prodrugs that are enzymatically transformed by GUK into the pharmacologically active substance (Miech et al., 1969; Elion, 1989). With the recovery of the cellular GDP pool, GUK regulates also the supply of guanine nucleotides to signal transduction pathways and cellular processes like protein synthesis or vesicular trafficking (Gaidarov et al., 1993; Brady et al., 1996).

Guanylate kinase regulation by RD3 might not be restricted to photoreceptor cells (Lavorgna et al., 2003; Azadi, 2013), since recent studies revealed that RD3 is also found in other tissue types like epithelial cells and is more ubiquitously expressed (Aravindan et al., 2017). Additionally, the authors suggest a metastasis suppressor function for RD3 in neuroblastoma (Khan et al., 2015). However, the underlying mechanisms and why RD3 is downregulated in tumor tissue is not clear so far. Ubiquitous expression and high affinity interaction of GUK and RD3 bring this protein complex more into focus.

## Author Contributions

HW, UJ-B and K-WK designed the study and planned experiments and wrote the manuscript. HW and UJ-B performed the experiments.

## Conflict of Interest Statement

The authors declare that the research was conducted in the absence of any commercial or financial relationships that could be construed as a potential conflict of interest.
