# Supplementary material for: Control of the Nucleotide Cycle in Photoreceptor Cell Extracts by Retinal Degeneration Protein 3
Source: Front Mol Neurosci. 2018 Feb 21;11:52. doi: 10.3389/fnmol.2018.00052 (PMC5826319; doi:10.3389/fnmol.2018.00052)
Supplement: Supplementary file 1 [file Image_1.PDF]

Supplementary information

Figure S1

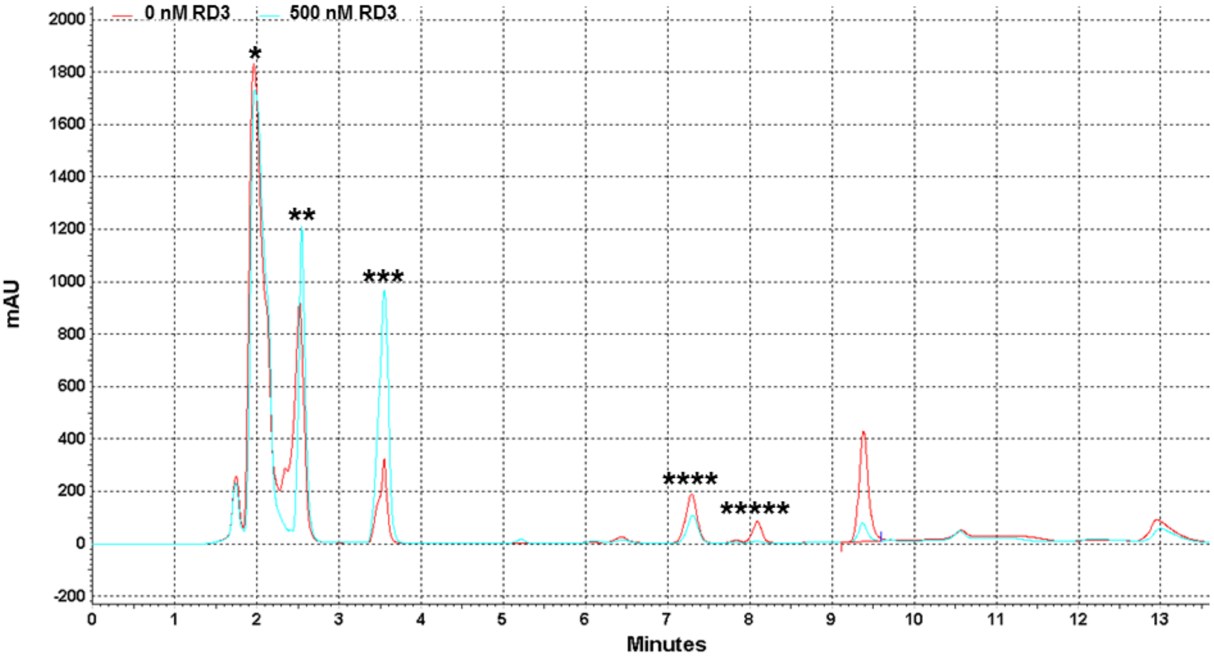

Figure S1: Example of a HPLC chromatogram for nucleotide analysis. GC-E activity was measured in washed bovine ROS membranes without RD3 present (red curve). The second measurement was done in the presence of 500 nM RD3 (blue curve). The retention time of different nucleotides was determined by using standards. Peaks refer to the following nucleotides: GTP (*), GDP (**), 5'-GMP (***), cGMP (****) and Guanosine (*****).

Figure S2

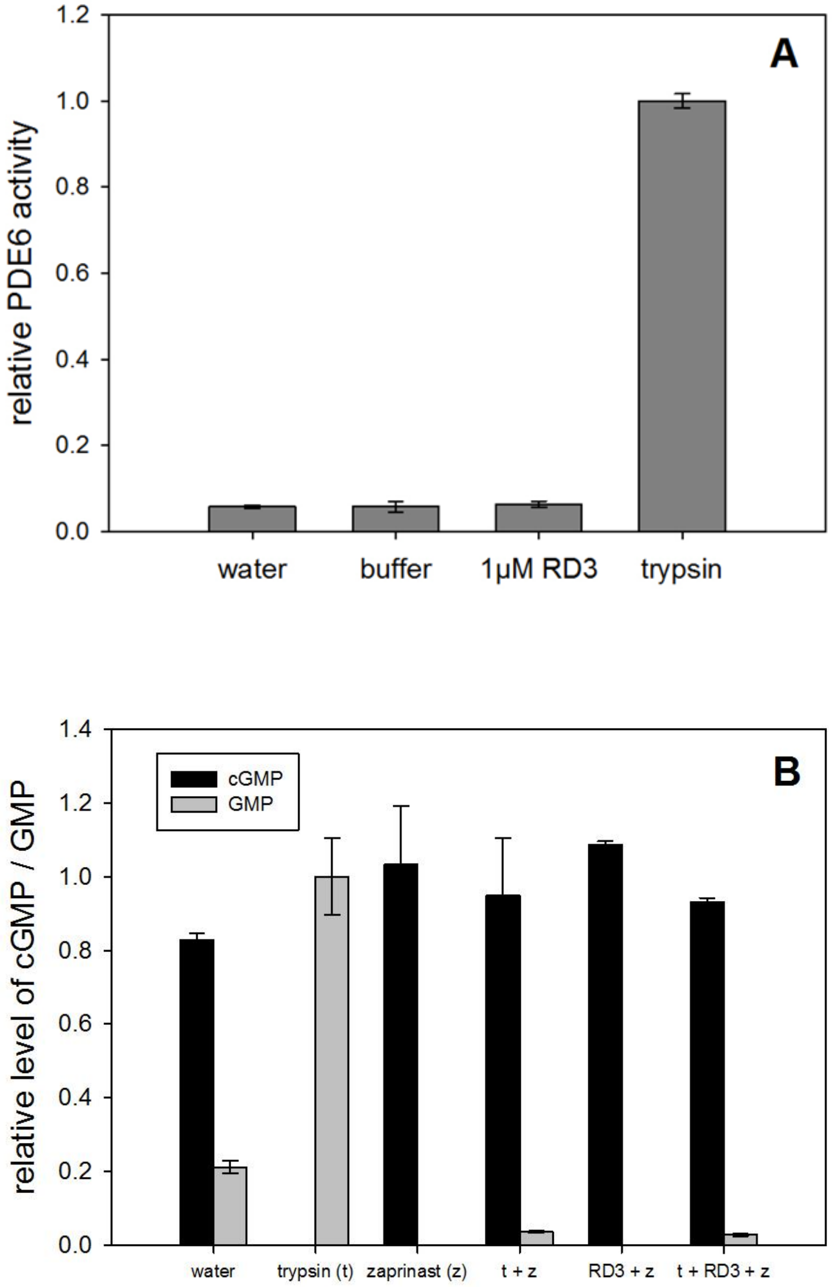

Figure S2: Activity of PDE6 in ROS samples. Hydrolysis product 5'-GMP of PDE activity was determined by HPLC analysis. (A) PDE6 activity was maximized by degradation of inhibitory PDE6 γ-subunits by trypsin, which was set to 100% activation. Control incubations of PDE6 were performed in water and buffer and for samples with RD3 added. (B) Effect of the PDE6 inhibitor zaprinast was tested. Trypsin leads to full activation whereby zaprinast blocks hydrolysis of 5'-GMP by PDE6. RD3 at a concentration of 1 µM was co-incubated with ROS samples and zaprinast.
